# Effects of vitamin D and omega-3 fatty acids co-supplementation on inflammatory biomarkers, tumor marker CEA, and nutritional status in patients with colorectal cancer: a study protocol for a double blind randomized controlled trial

**DOI:** 10.1186/s13063-019-3719-3

**Published:** 2019-12-09

**Authors:** Fatemeh Haidari, Behnaz Abiri, Masood Iravani, Seyed-Mohsen Razavi, Parvin Sarbakhsh, Kambiz Ahmadi-Angali, Mohammadreza Vafa

**Affiliations:** 1 Department of Nutrition, Nutrition and Metabolic Diseases Research Center, Ahvaz Jundishapur University of Medical Sciences, Ahvaz, Iran; 2 Department of Nutrition, Faculty of Paramedicine, Ahvaz Jundishapur University of Medical Sciences, Ahvaz, Iran; 3 Department of Oncology and Hematology, Faculty of Medicine, Tehran University of Medical Sciences, Tehran, Iran; 4 Department of Oncology and Hematology, Faculty of Medicine, Iran University of Medical Sciences, Tehran, Iran; 5 Department of Statistics and Epidemiology, School of Public Health, Tabriz University of Medical Sciences, Tabriz, Iran; 6 Faculty of Public Health, Ahvaz Jundishapur University of Medical Sciences, Ahvaz, Iran; 7 Department of Nutrition, School of Public Health, Iran University of Medical Sciences, Tehran, Iran; 8 Pediatric Growth and Development Research Center, Institute of Endocrinology and Metabolism, Iran University of Medical Sciences, Tehran, Iran

**Keywords:** Vitamin D, Omega-3 fatty acids, Colorectal cancer, Inflammatory biomarkers, Tumor marker CEA, Nutritional status

## Abstract

**Background:**

Much evidence is available demonstrating that both vitamin D and omega-3 fatty acids block the development and progression of colonic carcinogenesis. The results of animal studies have shown that the consumption of omega-3 fatty acids can decrease inflammatory biomarkers, enhance the efficacy of chemotherapy, and decrease the side effects of chemotherapy or cancer. Also, observational studies propose that higher levels of 25(OH)D are related to improved survival of colorectal cancer patients. This study will aim to evaluate the effects of vitamin D and omega-3 fatty acids co-supplementation on inflammatory biomarkers, tumor marker CEA, and nutritional status in colorectal cancer patients.

**Methods/design:**

We will carry out an 8-week double-blind randomized, placebo-controlled clinical trial to evaluate the effects of vitamin D and omega-3 fatty acids co-supplementation on inflammatory biomarkers, tumor marker CEA, and nutritional status in patients with stage ӀӀ or ӀӀӀ colorectal cancer undergoing chemotherapy.

**Discussion:**

Because of the important effects of vitamin D and omega-3 fatty acids on molecular pathways involved in cancer development and progression, it seems that both vitamin D and omega-3 fatty acids may provide a new adjuvant therapy by decreasing inflammatory biomarkers and resistance to cancer treatment in patients with colorectal cancer.

**Trial registration:**

Iranian Registry of Clinical Trials IRCT20180306038979N1. Registered on 16 March 2018.

## Background

Colorectal cancer (CRC) is an important public health problem and comprises a significant proportion of the global cancer burden with regard to morbidity and mortality. Epidemiological studies have demonstrated that CRC is the third most common cancer in the world [1], and it is ranked fourth in terms of deaths from cancer worldwide according to the World Health Organization [2]. Development of CRC is a multistep process including epigenetic and genetic changes that disturb cell homeostasis between cellular proliferation and apoptosis [1]. In colonic tumorigenesis, inflammatory cells lead to colitis by generating inflammatory factors and a variety of reactive oxygen species (ROS) and reactive nitrogen species (RNS). Oxidative stress impacts a large range of carcinogenic pathways because of its effects on DNA, RNA, proteins, and lipids, contributing to the progression of malignant transformation and proliferation of initiated cells [3]. Inflammation also enhances cancer progression by generating an inflammatory environment during tumor formation. The inflammatory and immunosuppressive agents released from these cells not only increase proliferation, angiogenesis, invasion, and metastasis, but also repress the host’s immune system and accelerate tumor growth and development of CRC [3]. Various molecular alterations occur during the development of CRC, including those involved in cell proliferation, cell survival, differentiation, resistance to apoptosis, metastasis, and tumor angiogenesis [4, 5].

It has been indicated that absolute carcinoembryonic antigen (CEA) level is an independent prognostic marker for CRC patients and may demonstrate tumor biological activity [6].

Throughout colonic carcinogenesis, particular molecular processes have been targeted for chemopreventive intervention, including chronic inflammation, proliferation, differentiation signaling, apoptosis, cell surface growth factor receptors, angiogenesis, and metastasis [3]. Despite understanding the pathway and mechanism of colon tumor progression, current therapies, including surgery, chemotherapy, radiotherapy, and molecular-targeted therapy, are still limited for some tumors [3]. Hence, a growing amount of scientific attention has been concentrated on evaluating the potential of dietary components for both prevention and control of CRC.

Among dietary components, a growing number of epidemiological, clinical, and experimental studies propose a protective impact of n-3 polyunsaturated fatty acids (PUFA; found in fish oil) on CRC [5, 7, 8]. Investigations with fish oil, which contains eicosapantaenoic acid (EPA; C20, omega-3) and docosahexaenoic acid (DHA; C22, omega-3), have demonstrated a protective impact against the induction and progression of experimentally generated colon cancer in an animal model [7]. Molecular mechanisms suggested to contribute to the multiple advantages of omega-3 fatty acids include: 1) suppression of the expression of cyclooxygenase-2 in malignant cells, thus reducing proliferation of these cells and consequently decreasing angiogenesis in them; 2) reduction of expression of AP-1 and ras, two oncogenes involved in tumor progression; 3) induction of differentiation in malignant cells; 4) repression of nuclear factor kappa-B (NF-kB) and bcl-2 expression, hence allowing apoptosis of cancer cells; and 5) decreased cancer-induced cachexia [8]. Moreover, dietary DHA significantly weakened the growth of human colon carcinoma WiDr in athymic mice containing mutated P53 [7]. The results of animal studies have shown that the intake of omega-3 fatty acids can slow the growth of cancer xenografts, promotes the efficacy of chemotherapy, and decreases the side effects of the cancer or of chemotherapy [7]. Therefore, it appears reasonable to consider that, along with cancer therapy, consumption of omega-3 fatty acids might reduce or stop the growth of metastatic malignant cells, enhance longevity of cancer patients, and improve their quality of life.

Several other reports have also demonstrated positive impacts of combining vitamin D or its analogues with a variety of chemotherapeutic factors in the treatment of various cancers [9–11]. Recently, supplementation with vitamin D analogues has been indicated to enhance the sensitivity of colon cancer cells to 5-fluorouacil and to increase the cytotoxic impacts of the drug both in vitro and in vivo [12–15]. The vitamin D receptor is present not only in cells and tissues involved in calcium regulation but also a large variety of other cells consisting of malignant cells [16]. Binding of vitamin D receptor by 1,25(OH)_2_D results in multiple cellular effects, including induction of differentiation and apoptosis and inhibition of proliferation, angiogenesis, and metastatic capability [17]. Remarkably, a relationship between the risk of developing cancer, latitude, low sun exposure, and low vitamin D has been reported [18]. Additional studies evaluating the relationship between serum 25(OH)D levels, dietary vitamin D intake, and cancer risk also observed a consistent reverse association between 25(OH)D concentration and CRC incidence [16, 17, 19]. Furthermore, the Women’s Health Initiative, a large randomized placebo controlled calcium/vitamin D trial, demonstrated that calcium plus vitamin D supplementation significantly reduced the risk of total, breast, and colorectal cancers [20]. Reverse relationships between serum 25(OH)D concentrations measured at diagnosis and subsequent CRC recurrence and mortality have also been reported [18]. On the other hand, the results of a nationwide, randomized, placebo-controlled trial of vitamin D_3_ (2000 IU per day) and marine omega-3 fatty acids (1 g per day) for the prevention of cancer and cardiovascular disease among men 50 years of age or older and women 55 years of age or older in the United States, demonstrated, during a median follow-up of 5.3 years, that supplementation with vitamin D or omega-3 fatty acids did not result in a lower incidence of invasive cancer than placebo [21, 22]. In addition, some meta-analyses looking at vitamin D and cancer incidence and mortality reported that vitamin D supplementation decreased the risk of cancer mortality [23, 24] but did not decrease total cancer incidence [24].

Given the importance of studying the role of vitamin D and omega-3 fatty acids as anticancer agents, and because the effect of co-supplementation with them on clinical outcomes of patients with CRC is not clear, the aim of this study is to evaluate the effects of vitamin D and omega-3 fatty acids co-supplementation on inflammatory biomarkers, tumor marker CEA, and nutritional status in patients with CRC.

## Methods/design

### Design

**Fig. 1:**
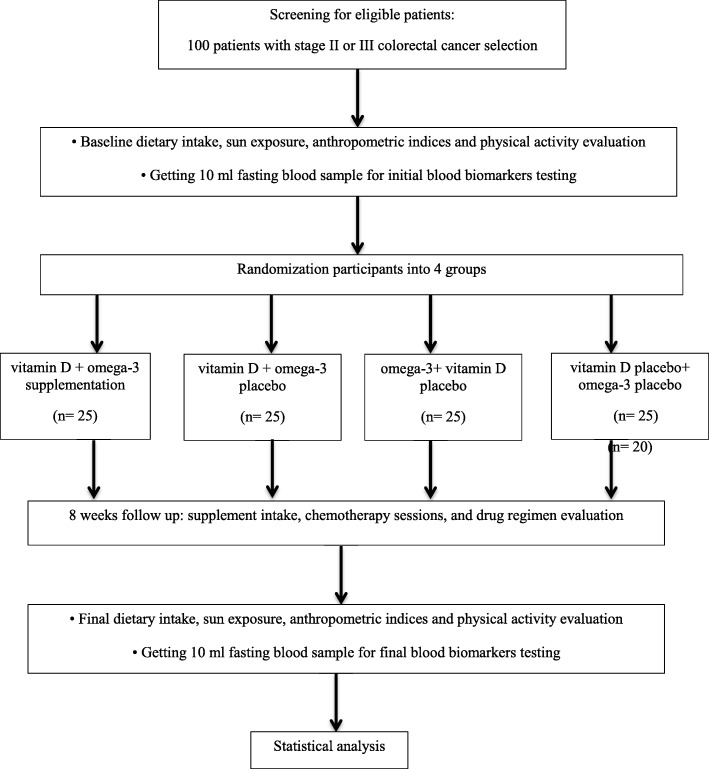
Protocol flow diagram; we will carry out an 8-week, double-blind, randomized controlled trial to determine the effects of vitamin D and omega-3 fatty acids co-supplementation on inflammatory biomarkers, tumor marker CEA, and nutritional status in patients with colorectal cancer

**Fig. 2:**
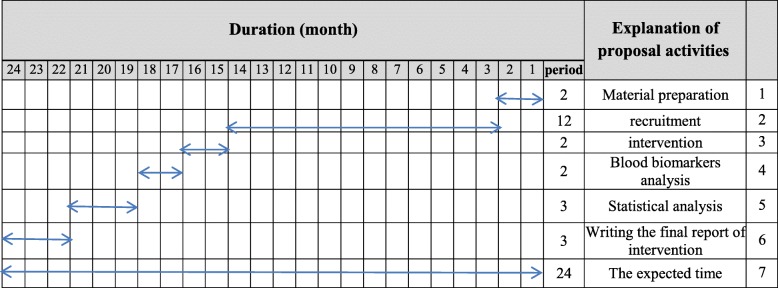
Timeline of the study; we expect the duration of this trial will be 24 months

We will conduct an 8-week, double-blind randomized controlled trial in a factorial design. This trial will be conducted at the oncology clinic of Tehran Gastroenterology & Hepatology Center (TGHC), Iran for 8 weeks to evaluate the effects of vitamin D_3_ (50,000 IU weekly) and omega-3 fatty acids (660 mg daily) co-supplementation on inflammatory biomarkers, tumor marker CEA, and nutritional status in patients with CRC (Figs. 1 and 2). The protocol is written in line with the Standard Protocol Items: Recommendations for Interventional Trials (SPIRIT) checklist (Additional file 1).

### Objectives and hypotheses of the study

The primary objective of the present study is to assess the impact of 8-week vitamin D_3_ and omega-3 fatty acids co-supplementation on serum 25(OH)D, inflammatory biomarkers, CRP/albumin, tumor marker CEA, body mass index (BMI), and body composition in patients with stage ӀӀ or ӀӀӀ CRC undergoing chemotherapy. As a second objective, the study will also evaluate the relationship between alterations in 25(OH)D levels and other variables. It is also expected that there will be improvement in evaluated variables.

### Participants

Participants will comprise 100 patients with stage II or III CRC. The inclusion criteria will consist of: patients with stage II or III CRC if they have to begin chemotherapy; aged > 18 years; BMI range of 18.5–30 kg/m^2^; serum 25(OH)D < 30 ng/ml; not having autoimmune disease, diabetes, hypertension, or renal, hepatic, parathyroid, and gastrointestinal disorders; not taking vitamin D and/or omeaga-3 supplements and other vitamin/mineral supplements or parenteral nutrition; not taking laxative and/or anti-inflammatory medications; not allergic to fish and fish products; and without history of having other cancers. Exclusion criteria will consist of: affected by any acute disease during the study; changes to the chemotherapy plan; refusal to continue with chemotherapy; unwilling to continue the study; also, we will exclude patients who were less than 90% compliant with treatment.

### Ethics and trial registration

CRC patients who meet the inclusion criteria will be completely informed about the protocol of the study. The protocol of this study was approved by the Medical Ethics Committee of Ahvaz Jundishapur University of Medical Sciences and is in conformity with the Declaration of Helsinki (approval number IR.AJUMS.REC.1396.1077). Each participant will sign the informed consent form. This clinical trial was registered on Iranian Registry of Clinical Trials (IRCT registration number IRCT20180306038979N1; http://irct.ir/user/trial/20288/view).

### Sample size

The number of participants was calculated according to the alterations in TNF-α between the control and co-supplementation groups and based on the study conducted by Mohammadzadeh et al. [25]. It was calculated with the use of fixed factor levels model (determining sample size for analysis of variance) [26] and considering sigma (standard deviation of TNF-α) of 1.25, 90% power, α of 0.05, resulting in 20 patients for each group. To allow for attrition, 25 patients will be recruited for each group. Collectively, a sample of 100 patients with stage II or III CRC will be enrolled.

### Randomization and blinding

The randomization assignment will be performed using computer-generated random numbers. The 100 participants who meet the criteria will be randomly allocated into four groups: 1) a 50,000 IU vitamin D soft gel (weekly) plus 2 omega-3 fatty acid capsules (daily), each capsule containing 330 mg omega-3 fatty acid; 2) a 50,000 IU vitamin D soft gel (weekly) plus 2 omega-3 fatty acid placebo (daily); 3) a vitamin D placebo (weekly) plus 2 omega-3 fatty acid capsules (each capsule containing 330 mg omega-3 fatty acid); and 4) a vitamin D placebo (weekly) plus 2 omega-3 fatty acid placebo (daily). Each omega-3 fatty acid capsule (MorDHA VISION, Minami Nutrition, Belgium) contains 54 mg EPA, 250 mg DHA, and 26 mg of other omega-3 fatty acids. The 50,000 IU vitamin D soft gels are supplied by Zahravi, Iran. The vitamin D and omega-3 fatty acid placebo contain oral paraffin and corn oil, respectively, are supplied by Zahravi, Iran, and are carefully matched in appearance with vitamin D and omega-3 fatty acid soft gels. Patients will receive verbal and written counseling on how to consume the capsules. Compliance will be evaluated by capsule count every 2 weeks. The supplementation will begin before the first session of chemotherapy.

For blinding, a person not involved in the study protocol will create the randomization list, assigning participants to the vitamin D, omega-3, co-supplementation, or placebo group. Vitamin D, omega-3, and placebo tablets will be placed into unlabeled identical containers. The study leader will label these containers with participant numbers using the randomization list. All investigators and participants will be blinded to the random assignments.

### Measurements

A questionnaire about patients’ medications, diseases, cancer history, and probable supplement use will be recorded at the beginning of the intervention. Dietary intake will be evaluated by 3-day, 24-h record questionnaires (two week days and one weekend day) at baseline and the end of the intervention, and total energy, macronutrient, and some micronutrient (vitamins A, D, E; calcium and selenium) intake will be estimated using nutritionist IV software. To evaluate physical activity levels the short form of the international physical activity questionnaire (IPAQ) [27] will be used at baseline and the end of the intervention. Sun exposure will be assessed with the questionnaire [28] at baseline and the end of the study. A sunshine exposure score is obtained based on (a) how often and what time of day the subjects are outside, (b) which parts of the body are exposed as a percentage of total body surface area, and (c) do the subjects use sun screen or not. Anthropometric indices will be measured after overnight fasting, with minimal clothing and without shoes, before and after the intervention. Body weight will be measured to an accuracy of 0.1 kg using a Beurer scale (Beurer, Germany). Height will be assessed to the nearest 0.5 cm in the standing position without shoes. BMI will be computed as body weight (kg) divided by height squared (cm^2^). A bioelectric impedance analyzer (Quad scan 4000; Body stat) will be used to measure total body fat and fat free mass percentage. The difference in CRP/albumin levels, as an indicator of nutritional status, between groups will also be compared with score values proposed by Correa et al.: no risk ≤ 0.4; low risk = 0.4–1.2; medium risk = 1.2–2; high risk ≥ 2.0 [29]. This indicator will be assessed at baseline and end of the intervention.

Before and after the intervention, blood samples will be collected after 12-h overnight fasting. The serum of patients will be kept at − 80 °C until biochemical analyses. Serum 25(OH)D will be measured by enzyme-linked immunosorbent assay (ELISA) and Euro Immun kit (Euro Immun, Germany). Serum TNF-α, IL-1β, IL-6, and IL-8 levels will be assessed by ELISA and Bender Med kit (Bender Med, Germany). Serum CRP and albumin will be determined by ELISA and Binding kit (Binding, UK). Tumor marker CEA will be evaluated by ELISA and CanAg kit (CanAg, Italy).

### Statistical analysis

All analyses will be performed with STATA statistics software, version 11.0 (STATA Corporation, College Station, EUA). Kolmogorov-Smirnov test will be used to assess normality of data. Quantitative and normally distributed parameters will be expressed as mean + standard deviation. Quantitative and non-normally distributed data will be shown as median and interquartiles. Analysis of variance (ANOVA) test will be used for comparing the values between and within groups. Analysis of covariance (ANCOVA) will be used to identify any differences between the four groups at the end of study, adjusting for baseline values and covariates (such as dietary intake, physical activity, and sun exposure). Bonferroni will be used to pairwise comparisons for the values after the intervention.

## Discussion

Vitamin D metabolites exert significant anti-neoplastic effects in preclinical models. In clinical trials, the effect of vitamin D has been reported in various types of cancer. A low vitamin D level and/or activity is related to an increased risk of cancer and a more aggressive tumor growth, while high activity of this pathway leads to anti-tumoral impacts [2]. Moreover, some meta-analyses looking at vitamin D and cancer incidence and mortality reported that vitamin D supplementation decreased the risk of cancer mortality [23, 24].

Previous investigations in health subjects have reported a relationship between polyunsaturated long chain n-3 fatty acids and lower levels of pro-inflammatory biomarkers and the tumor marker CEA, supporting the concept that n-3 fatty acids may be useful in patients affected by diseases described by active inflammation.

Inflammation is a common feature of cancer. The presence and extent of a chronic systemic inflammatory response may lead to progressive nutritional decline. Therefore, a specific nutritional intervention resulting in a decrease in the inflammatory state, and consequently ameliorating nutritional status, could be a successful strategy for CRC therapy.

Based on the results of previous studies, it seems that both vitamin D and omega-3 fatty acids may provide a new complementary treatment by decreasing inflammatory biomarkers and resistance to cancer treatment in patients with CRC. The question remains whether vitamin D and omega-3 fatty acids co-supplementation would actually be useful after diagnosis of CRC. So, the aim of this study is to investigate the effects of vitamin D and omega-3 fatty acids co-supplementation on inflammatory biomarkers, tumor marker CEA, and nutritional status in patients with CRC.

### Trial status

This trial is in the enrolment stage.

Protocol version 4, 5/4/2019.

Date recruitment began on 4/30/2018.

Recruitment will be completed on 5/15/2019.

## Supplementary information

**Additional file 1.** SPIRIT 2013 checklist: Recommended items to address in a clinical trial protocol and related documents*.

## Data Availability

Not applicable.

## Abbreviations

BMI: Body mass index
CEA: Carcinoembryonic antigen
CRC: Colorectal cancer
IPAQ: International physical activity questionnaire
RNS: Reactive nitrogen species
ROS: Reactive oxygen species
