# Supplementary material for: Effects of vitamin D and omega-3 fatty acids co-supplementation on inflammatory biomarkers, tumor marker CEA, and nutritional status in patients with colorectal cancer: a study protocol for a double blind randomized controlled trial
Source: Trials. 2019 Dec 9;20:682. doi: 10.1186/s13063-019-3719-3 (PMC6900845; doi:10.1186/s13063-019-3719-3)
Supplement: Supplementary file 1 — Additional file 1. SPIRIT 2013 checklist: Recommended items to address in a clinical trial protocol and related documents*. [file 13063_2019_3719_MOESM1_ESM.doc]

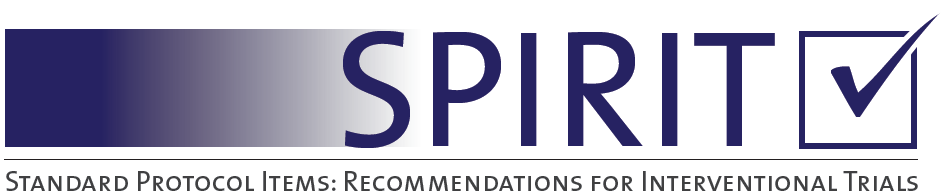


SPIRIT 2013 Checklist: Recommended items to address in a clinical trial protocol and related documents*

| Section/item | ItemNo | Description |
| --- | --- | --- |
| **Administrative information** | | |
| Title | 1 | Title, page: 1 |
| Trial registration | 2a | In abstract and methods, page: 3 |
| Protocol version | 3 | 3 |
| Funding | 4 | In declaration, page: 11 |
| Roles and responsibilities | 5a | In title page and declaration, page: 1 and 11 |
| Introduction |  |  |
| Background and rationale | 6a | Background, page: 4 |
| Objectives | 7 | Objectives and hypotheses of the study, page: 6 |
| Trial design | 8 | Method, page: 6,7,8 |
| Methods: Participants, interventions, and outcomes | | |
| Study setting | 9 | Method: page 6,7 |
| Eligibility criteria | 10 | Method, page: 6,7 |
| Interventions | 11a | Method. Page: 7 |
| Outcomes | 12 | Method. Page: 7,8 |
| Participant timeline | 13 | Figure 2. page 16 |
| Sample size | 14 | Method/sample size. Page: 7 |
| Recruitment | 15 | Method/sample size. Page: 7 |
| **Methods: Assignment of interventions (for controlled trials)** | | |
| Allocation: |  |  |
| Sequence generation | 16a | Method/randomization. Page: 7,8 |
| Allocation concealment mechanism | 16b | Method/randomization and blinding. Page: 7,8 |
| Implementation | 16c | Method/randomization and blinding. Page: 7,8 |
| Blinding (masking) | 17a | Method/randomization and blinding. Page: 7,8 |
|  | 17b | Method/randomization and blinding |
| **Methods: Data collection, management, and analysis** | | |
| Data collection methods | 18a | Method, page: 8,9 |
| Data management | 19 | Method, page: 8 |
| Statistical methods | 20a | Method, page: 9 |
|  |  | |
| **Methods: Monitoring** | | |
| Data monitoring | 21a | N/A |
|  | 21b | N/A |
| Harms | 22 | N/A |
| Auditing | 23 | N/A |
| Ethics and dissemination | | |
| Research ethics approval | 24 | Method and declaration, page: 10,11 |
| Protocol amendments | 25 | N/A |
| Consent or assent | 26a | Method, page: 7 |
|  | 26b | N/A |
| Confidentiality | 27 | N/A |
| Declaration of interests | 28 | N/A |
| Access to data | 29 | N/A |
| Ancillary and post-trial care | 30 | N/A |
| Dissemination policy | 31a | N/A |
|  | 31b | N/A |
|  | 31c | N/A |
| Appendices |  |  |
| Informed consent | 32 | Method, page: 7 |
| Biological specimens | 33 | N/A |

*It is strongly recommended that this checklist be read in conjunction with the SPIRIT 2013 Explanation & Elaboration for important clarification on the items. Amendments to the protocol should be tracked and dated. The SPIRIT checklist is copyrighted by the SPIRIT Group under the Creative Commons “[Attribution-NonCommercial-NoDerivs 3.0 Unported](http://www.creativecommons.org/licenses/by-nc-nd/3.0/)” license.
